# Analyzing Heart Rate Variability for COVID-19 ICU Mortality Prediction Using Continuous Signal Processing Techniques

**DOI:** 10.3390/jcm14155312

**Published:** 2025-07-28

**Authors:** Guilherme David, André Lourenço, Cristiana P. Von Rekowski, Iola Pinto, Cecília R. C. Calado, Luís Bento

**Affiliations:** 1 ISEL—Instituto Superior de Engenharia de Lisboa, Instituto Politécnico de Lisboa, Rua Conselheiro Emídio Navarro 1, 1959-007 Lisbon, Portugalarl@cardio-id.com (A.L.); cecilia.calado@isel.pt (C.R.C.C.); 2 NOVA LINCS—NOVA Laboratory for Computer Science and Informatics and CardioID Technologies, 2829-516 Caparica, Portugal; 3 NMS—NOVA Medical School, FCM—Faculdade de Ciências Médicas, Universidade NOVA de Lisboa, Campo Mártires da Pátria 130, 1169-056 Lisbon, Portugal; 4 CHRC—Comprehensive Health Research Centre, Universidade NOVA de Lisboa, 1150-082 Lisbon, Portugal; 5 NOVA Math—Center for Mathematics and Applications, NOVA FCT—NOVA School of Science and Technology, Universidade NOVA de Lisboa, Campus da Caparica, 2829-516 Caparica, Portugal; 6 iBB—Institute for Bioengineering and Biosciences, i4HB—The Associate Laboratory Institute for Health and Bioeconomy, IST—Instituto Superior Técnico, Universidade de Lisboa, Av. Rovisco Pais, 1049-001 Lisbon, Portugal; 7 Intensive Care Department, ULSSJ—Unidade Local de Saúde de São José, Rua José António Serrano, 1150-199 Lisbon, Portugal; 8 Integrated Pathophysiological Mechanisms, CHRC—Comprehensive Health Research Centre, NMS—NOVA Medical School, FCM—Faculdade de Ciências Médicas, Universidade NOVA de Lisboa, Campo Mártires da Pátria 130, 1169-056 Lisbon, Portugal

**Keywords:** HRV, mortality, ICU, COVID-19

## Abstract

**Background/Objectives:** Heart rate variability (HRV) has been widely investigated as a predictor of disease and mortality across diverse patient populations; however, there remains no consensus on the optimal set or combination of time and frequency domain nor on nonlinear features for reliable prediction across clinical contexts. Given the relevance of the COVID-19 pandemic and the unique clinical profiles of these patients, this retrospective observational study explored the potential of HRV analysis for early prediction of in-hospital mortality using ECG signals recorded during the initial moments of ICU admission in COVID-19 patients. **Methods:** HRV indices were extracted from four ECG leads (I, II, III, and aVF) using sliding windows of 2, 5, and 7 min across observation intervals of 15, 30, and 60 min. The raw data posed significant challenges in terms of structure, synchronization, and signal quality; thus, from an original set of 381 records from 321 patients, after data pre-processing steps, a final dataset of 82 patients was selected for analysis. To manage data complexity and evaluate predictive performance, two feature selection methods, four feature reduction techniques, and five classification models were applied to identify the optimal approach. **Results:** Among the feature aggregation methods, compiling feature means across patient windows (Method D) yielded the best results, particularly for longer observation intervals (e.g., using LDA, the best AUC of 0.82±0.13 was obtained with Method D versus 0.63±0.09 with Method C using 5 min windows). Linear Discriminant Analysis (LDA) was the most consistent classification algorithm, demonstrating robust performance across various time windows and further improvement with dimensionality reduction. Although Gradient Boosting and Random Forest also achieved high AUCs and F1-scores, their performance outcomes varied across time intervals. **Conclusions:** These findings support the feasibility and clinical relevance of using short-term HRV as a noninvasive, data-driven tool for early risk stratification in critical care, potentially guiding timely therapeutic decisions in high-risk ICU patients and thereby reducing in-hospital mortality.

## 1. Introduction

The autonomic nervous system (ANS) regulates different involuntary mechanisms essential for maintaining internal homeostasis, including the function of organs, tissues, and metabolic systems. It is divided into the sympathetic and parasympathetic systems, which act in opposition. The sympathetic system prepares the body for action, activating the cardiovascular system and raising heart rate, cardiac output, contractility, and blood pressure. Conversely, the parasympathetic system promotes rest and recovery by decreasing these functions as a way to store energy [1]. Dysregulation of either branch is associated with various diseases, with sympathetic overactivity notably contributing to conditions such as hypertension, congestive heart failure, cardiac tachyarrhythmias, and sudden cardiac death [1,2].

A way of assessing autonomic function is by analyzing heart rate variability (HRV), which is the variation in time intervals between consecutive heartbeats using an electrocardiogram (ECG). HRV captures the complex, dynamic interplay between the heart and brain through nonlinear autonomic processes, serving as a marker of neurocardiac regulation and adaptability to different internal and external stimuli. As such, it is influenced by factors such as blood pressure, gas exchange, vascular tone, and gastrointestinal activity [3]. Adequate HRV is synonymous with good adaptability, characterizing healthy individuals with efficient compensatory mechanisms, whereas low HRV is often associated with abnormal and insufficient adaptation of the autonomic nervous system. Therefore, changes in HRV patterns can be used to assess patients’ health status [3].

**Figure 1:**
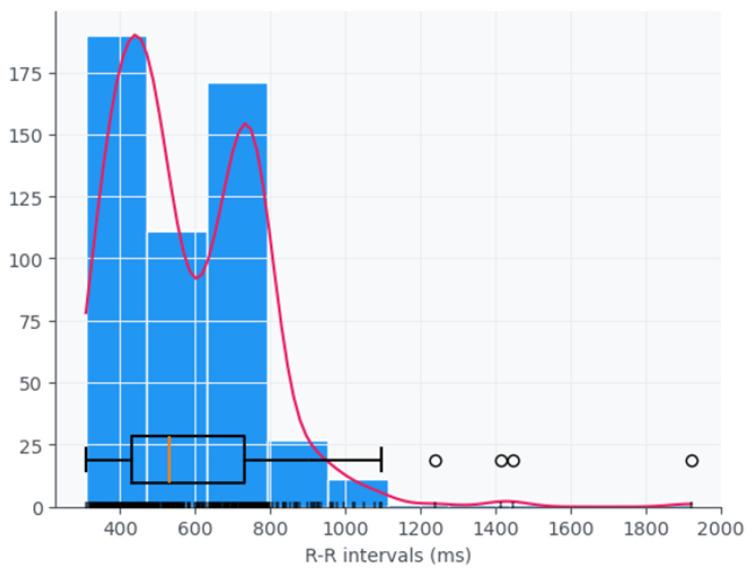
Histogram of absolute frequencies, illustrating distribution of RR intervals in a 2 min space, using the data from our study.

Despite there still not being a consensus in the literature on the optimal set of features or the most effective combination of parameters to quantify HRV—likely because all are calculated from RR intervals and often show a significant degree of correlation—various kinds of information can be used [4]. This includes time-domain, frequency-domain, and nonlinear measures, each offering distinct perspectives on autonomic function. Time-domain indices quantify the variability in successive heartbeat intervals, reflecting fluctuations in the duration of cardiac cycles. Their analysis relies on statistical methods, among which the most used are calculated from RR intervals (Figure 1 in the ECG). The most common are the standard deviation of normal RR intervals (SDNN), representing overall variability within a given time window, and the root mean square of successive differences (RMSSD), which captures short-term variability and is a marker of parasympathetic activity.

Frequency-domain parameters estimate power distribution across four main frequency bands—ultra-low (ULF), very low (VLF), low (LF), and high frequency (HF)—although other bands may be analyzed depending on recording duration. The ULF band (≤0.003 Hz) reflects RR interval fluctuations over 5 min to 24 h; VLF (0.0033–0.04 Hz) comprises rhythms with 25–300 s periods; LF (0.04–0.15 Hz) corresponds to 7–25 s cycles and is influenced by respiration; and HF (0.15–0.40 Hz), or the respiratory band, is more strongly affected by respiration. The LF/HF ratio is often used to estimate the balance between sympathetic and parasympathetic nervous system activity under controlled conditions [3,5,6]. Finally, nonlinear methods assess the unpredictability of a time series, reflecting the complexity of HRV regulation, thereby needing a larger number of RR intervals. These methods have been increasingly applied to predict the behavior of biological phenomena, proving to be strong mortality predictors. Among the most used nonlinear approaches are heart rate asymmetry, heart rate fragmentation, and the Poincaré plot [7,8]. The NeuroKit2 library [9] provides a total of 91 HRV indices, including time-domain, frequency-domain, and nonlinear measures. An overview of these parameters is presented in Table 1, with detailed descriptions available in Supplementary Table S1.

In this article, we focus our study on the use of HRV as a predictor of ICU mortality in COVID-19 patients. Prior research has demonstrated its potential in other critical care contexts. For example, using five-minute Holter ECG segments from 55 patients, Bishop et al. [10] focused on the frequency domain and found that reduced VLF power was significantly associated with 30-day mortality (*OR* = 0.6, 95% confidence interval (CI): 0.396–0.911, *p* = 0.016). Moridani et al. [11] reported that nonlinear recurrence quantification analysis (RQA) parameters were more sensitive to physiological decline than traditional HRV metrics like LF/HF and SD2/SD1 ratios, and significant changes in RQA measures were observed even in episodes further from death. Liu et al. [12] focused on a different approach, exploring measures of heart rate n-variability (HRnV) to predict 30-day hospital mortality in 66 septic patients, showing that HRnV-based models improved predictive accuracy (AUC = 0.77, 95% CI: 0.70–0.84) compared to models using only vital signs and conventional HRV. Indeed, many studies have already suggested the usefulness of HRV measures in predicting outcomes in critically ill patients with different clinical conditions. However, there is still limited information on how HRV relates specifically to COVID-19 in the ICU, despite the severe impact of the disease during the pandemic [13,14].

Since viral infections can affect the ANS, it has been proposed that SARS-CoV-2 may also be involved in ANS dysfunction. This is supported by observations of irregular heart rhythms and blood pressure changes in patients with COVID-19 [15]. SARS-CoV-2 can activate the sympathetic nervous system and elicit an inflammatory response, with the vagus nerve playing a central role in modulating this process. As a key neuroimmunomodulator, the vagus nerve communicates peripheral inflammation to the brain and reflexively inhibits it. Mol et al. [14] explored the prognostic value of HRV, hypothesizing that inadequate vagal inhibition of the immune response may influence the risk of adverse outcomes—an effect measurable through HRV analysis. Their study analyzed 10-second, single 12-lead ECG recordings from 271 patients at hospital admission, using SDNN to predict 3-week survival and ICU admission. The results showed that lower HRV was predictive of ICU admission within the first week (HR = 0.51, 95% CI: 0.29–0.90), highlighting its potential as a noninvasive marker for risk stratification in COVID-19 patients. Their findings also emphasised the relationship between reduced HRV and vagus nerve dysfunction, which may contribute to the hyperinflammation and ARDS observed in more severe COVID-19 cases. Komaenthammasophon et al. further supported this connection by linking decreased HRV to elevated inflammatory markers and higher mortality in 65 ICU COVID-19 patients. Among the biomarkers, high-sensitivity C-reactive protein showed the most significant increase in association with lower HRV, while reduced SDNN values (the only HRV parameter that was calculated) were most strongly correlated with mortality [13].

As discussed and supported by the existing literature, HRV tends to vary inversely with clinical severity and patients’ prognosis in the ICU, regardless of the underlying condition [16]. However, the methodologies, extracted parameters, and modeling techniques used to assess HRV vary significantly. While time-domain features like SDNN reflect both vagal and sympathetic activity, and thereby are more commonly used in COVID-19 research, other valuable parameters like RMSSD which also capture the vagal tone [14] remain underexplored. Moreover, some studies focus on frequency-domain analysis, while others incorporate nonlinear methods, suggesting that combining multiple domains may enhance predictive performance. To address these gaps, this study adopts a more extensive approach by extracting 83 HRV features across time (19 features), frequency (8 features), and nonlinear (56 features) domains. By applying four feature reduction methodologies and five different classification models, the goal of this article is to identify the most informative combinations of HRV features for accurately predicting ICU mortality in COVID-19 patients.

## 2. Materials and Methods

This is a retrospective observational study involving COVID-19 patients admitted to the ICU at Hospital de São José, a tertiary care facility in Lisbon, between 24 June 2020 and 16 February 2022.

The data collection process complied with all legal and ethical requirements, including patient anonymity, written informed consent, and approval by the hospital’s Ethics Committee (1043/2021, 20 May 2020). The study was conducted within the scope of the Predictive Models of COVID-19 Outcomes for Higher Risk Patients Towards a Precision Medicine (PREMO) project.

COVID-19 diagnosis was confirmed with real-time polymerase chain reaction tests for SARS-CoV-2. After collecting medical records and files of all patients admitted to the ICU for COVID-19 during the specified period, individuals under 18 years of age were excluded. The inclusion criteria were defined in the pre-processing section of the methodology.

Regarding the patients’ baseline demographic characteristics, age categories were defined according to the World Health Organization (WHO) criteria for elderly individuals. COVID-19 waves were established based on the pandemic timeline in Portugal using the dates reported in Von Rekowski et al. [17]. Obesity was defined according to the WHO guidelines as a BMI of 30.0 or higher. In cases where the BMI was not available, patients’ clinical medical records were reviewed to confirm a history of obesity. Respiratory support included the need for invasive mechanical ventilation (IMV) and/or extracorporeal membrane oxygenation (ECMO) at any time during ICU admission.

**Figure 2:**
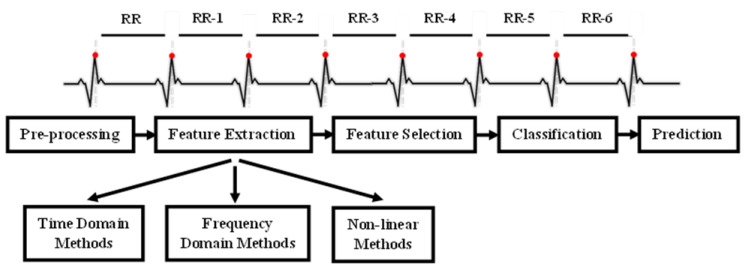
HRV analysis methodology: from raw physiological data to automated interpretation. The top panel illustrates RR interval detection from the ECG signal, which forms the basis for feature extraction. The bottom panel outlines the subsequent steps of the HRV analysis pipeline.

In the following subsections, we outline the main tools and methodologies used in the study. The overall methodology follows the pipeline depicted in Figure 2. We begin with data presentation and pre-processing steps, followed by the signal analysis methodologies and the classification models used to predict patient mortality. All the pipeline was implemented in Python 3.12 using Numpy, Matplotlib, Scipy, and Scikit-learn 1.7 [18].

### 2.1. Pre-Processing

The analysis conducted in this study relied on physiological signal data collected from ICU patients. Prior to the development of predictive models, it was necessary to explore and structure the available data to enable meaningful interpretation and consistent processing.

**Figure 3:**
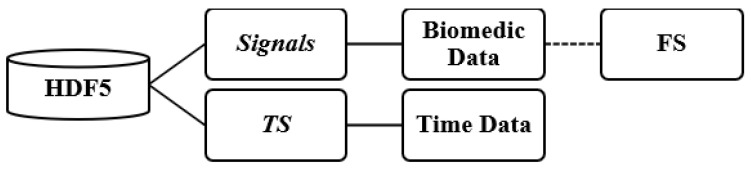
Hierarchy of the HDF5 files used in this study. Each file contains signals, storing the biomedical waveforms along with their sampling frequencies, and TS, containing the corresponding timestamp data. The biomedic data and time data entries reflect the actual recorded signals and their temporal alignment, respectively. FS refers to the sampling frequency associated with each signal.

The patient files containing the recorded signals were stored in HDF5 format [19]. The file structure was organized into two main groups: signals and timestamp (TS) (see Figure 3). The signals group included a range of physiological signals collected during patients’ hospitalizations, along with their respective sampling frequencies (FS), typically set at either 128 Hz or 256 Hz.

Each recording corresponded to a continuous monitoring period and included a variable number of signals, depending on the clinical apparatus connected to the patient at the time. The majority of the records included ECG leads I, II, III, and aVF, along with photoplethysmogram, intra-arterial blood pressure, and respiratory waveforms. In some cases, additional signals such as capnography and ECG leads aVL and aVR were also available.

Each file was organized according to hospital bed assignment and defined time intervals. A single file could contain multiple recordings, sometimes from different patients and hospitalizations. In many cases, data from multiple ICU admissions were merged into a single file. This partial association reflected the logistical challenges and systemic disarray experienced by hospitals during the COVID-19 pandemic. The duration of the files varied widely, with maximum duration being approximately ten days and the minimum duration around one hour.

The dataset used in this study comprises 381 records associated with 321 patients, indicating that some patients had more than one entry. All this information is distributed across 245 individual files.

A pre-processing step was essential to isolate each patient recording. This involved identifying transitions between patients by detecting prolonged gaps in the signal or TS data, which could be recognized through signal and/or TS plots. In the TS, interruptions in the data recording were identified by abrupt changes in the signal slope, marking the end and beginning of different recording segments. However, in some cases, monitoring systems were not reset between patient transitions, and as a result, these changes were not reflected in the TS data. Such instances were identified through visual inspection of the biomedical signals, where patient changes without system restarts appeared as prolonged periods with near-zero slope. It is important to note that each biomedical signal was recorded independently, leading to variability in the number and duration of detected interruptions across signals.

To accurately separate patient records, a minimum duration threshold was defined, as many brief temporal gaps did not represent actual patient transitions. These short gaps were often not repeated across the other signals and were therefore excluded. Only points where the difference to the subsequent point exceeded twenty minutes were retained, forming intervals. Additionally, gaps shorter than three seconds were merged to avoid unnecessary fragmentation of a single interval into multiple segments.

Using signal overlap analysis, interruptions that were simultaneously present in all TS signals and all biomedical signals were identified. The start and end points of each interval were defined to represent the smallest region of concurrent interruption in all signals. These intervals were saved and later matched with clinical metadata to determine which ones corresponded best to the documented ICU admissions.

**Figure 4:**
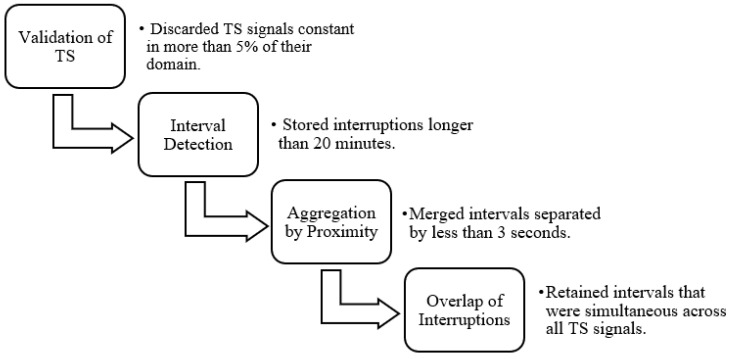
Detection of the different records in each file.

Figure 4 illustrates the steps followed for data validation and for the detection of individual hospitalizations in each record.

The extracted signal segments were then categorized according to their need for additional processing. For each hospitalization, a list of all validated time intervals was generated. These intervals were matched against ICU admission episode numbers and discharge dates recorded in the clinical database to accurately assign the biomedical signals to their corresponding ICU admissions. Discrepancies between the registered ICU admission and discharge dates and those detected by the algorithm were individually analyzed. Hence, cases in which the ICU admission dates did not align with the start of the signal segments identified by the algorithm, as well as those involving patient readmission, were excluded. After the separation of ICU admissions was complete, the next step was to select in each file the segments that would be used to extract features. Since this study focuses on the initial moments of ICU admission (the first 15, 30, and 60 min), files that did not contain data from this period were excluded. Furthermore, files were excluded if the discrepancy between the admission to the ICU identified by the algorithm and the one on the patient’s chart exceeded 3 h. In cases of patient readmission, only the first admission record was considered.

Following this selection process, a total of 82 valid records were retained, each corresponding to a unique patient. A brief description of these patients’ demographic and clinical characteristics can be observed in Table 2. A large percentage of the patients were elderly (46.3%), and most were male, which is typical for COVID-19 cases. Only a small proportion of patients belonged to the first wave of the pandemic, likely because ICUs were overwhelmed at that time, and the data signal was difficult to extract—during the first wave, there were more overlapping patient records within single files, as well as less systematic record-keeping. Most patients had between 1 and 3 comorbidities, with arterial hypertension, dyslipidemia, and diabetes being the most frequent; these are common comorbidities in Portugal, especially considering the patients’ median age was 57 years. A significant percentage required IMV, a common feature related to ARDS in COVID-19 patients, which was the most frequent admission motive (47.6%). In total, there were 66 discharges and 16 deaths.

Due to variability in signal acquisition, not all ECG leads were consistently recorded across all patients. Consequently, the number of patients included in the final analysis varied depending on the specific lead under consideration. The distribution of patients per lead, distinguishing between discharges and deaths, is presented in Table 3. The rationale for retaining data from all four leads was to enable performance comparisons across them, providing insight into how lead selection may influence the extracted HRV features. By evaluating model performance using different leads, the study also aimed to assess the adaptability of the models to varying ECG configurations commonly encountered in clinical settings.

### 2.2. Feature Extraction, Selection, and Classification

For the computation of HRV features, various time window lengths have been used in the literature, typically selected based on the dynamics of the patient or user. In the ICU, due to the fast dynamics of physiological signals, shorter time windows are generally preferred [20]. In this study, we selected window lengths of 2, 5, and 7 min, applying sliding windows with a 50% overlap between consecutive segments. These window lengths will be referred to as intervals, denoted as inter2, inter5, and inter7, respectively. The signal analysis was limited to the initial phase of ICU admission, with mortality predictions based on data recorded during the first 15, 30, and 60 min/time periods (T).

**Figure 5:**
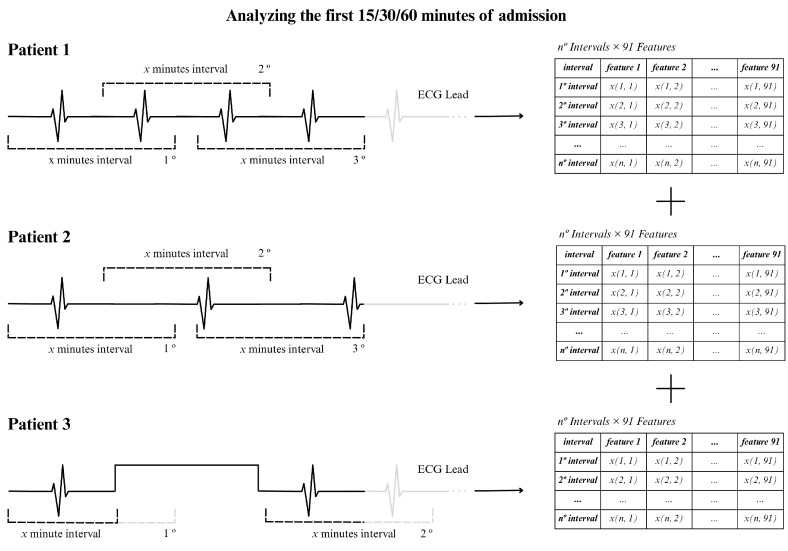
Example for the 15 min signal analysis.

To illustrate the procedure, we show an example in Figure 5, using an ECG signal of 15 min and using 2 min windows. In this situation, approximately 13 windows were generated assuming that the RR intervals were extracted continuously, as for patients 1 and 2 in the figure (top and center). For patient 3 (bottom part of the figure) there was an acquisition interruption, which led to the presence of fewer windows of 2 min. In general, the actual number of windows varied between patients due to interruptions in individual signal recordings, resulting in different window counts for the 15, 30, or 60 min time periods.

Each record was processed individually, beginning with the detection of R-peaks, using the Hamilton approach [21] and computation of the RR time series. From there, a total of 91 features were computed, including 25 time-domain features, 10 frequency-domain features, and 56 nonlinear domain features (7 indices derived from the Poincare plot, 4 heart rate fragmentation parameters, 16 heart rate asymmetry indices, and 29 indices related to complexity and fractal physiology). However, only 83 features were retained for analysis, as some could not be calculated for the selected window sizes.

For feature reduction, several approaches were employed: (i) variance thresholding—simple unsupervised method that removes all features whose variance does not meet some threshold; (ii) k-best selection—selects the 10 best features ranked by the ANOVA F-value between label/feature; (iii) tree-based feature selection—based on impurity criteria [22]; (iv) and a SHapley Additive explanation (SHAP)-based selection [23]—rooted in cooperative game theory and used to evaluate the importance of prediction parameters in the model. This combination of methods aimed to identify the most relevant set of characteristics to predict ICU mortality.

Following feature selection, two distinct approaches were applied to aggregate the information of each window length, namely, Method C (Consensus) and Method D (Decreased).

**Figure 6:**
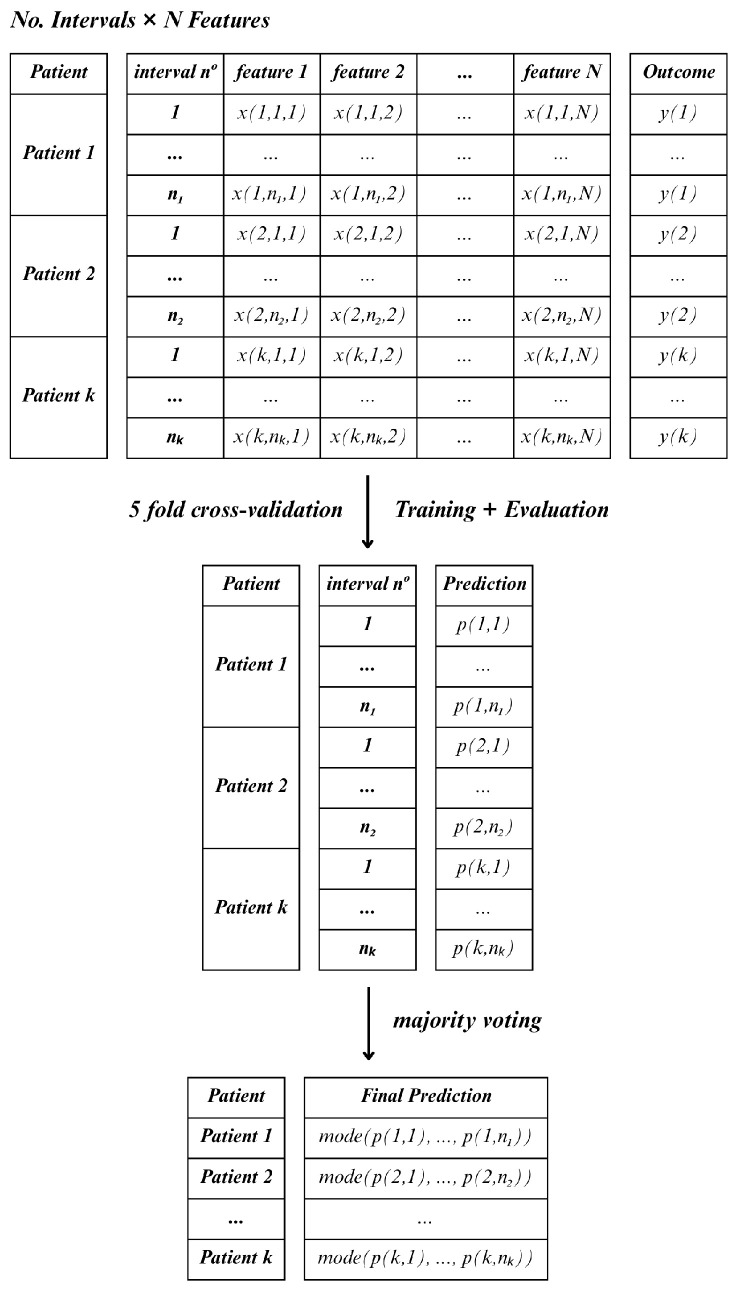
Method C—majority voting approach.

In Method C, for each lead and hospitalization period (15, 30 and 60 min), all feature observations were retained across all windows within the specified interval, treating each window as an individual sample. As a result, a single patient could contribute multiple instances, which would mean that the total number of samples would exceed the actual number of patients. In this case, the classifier was applied to each sample independently, and final patient-level predictions were determined using a majority voting scheme (the mode) across all windows Figure 6.

**Figure 7:**
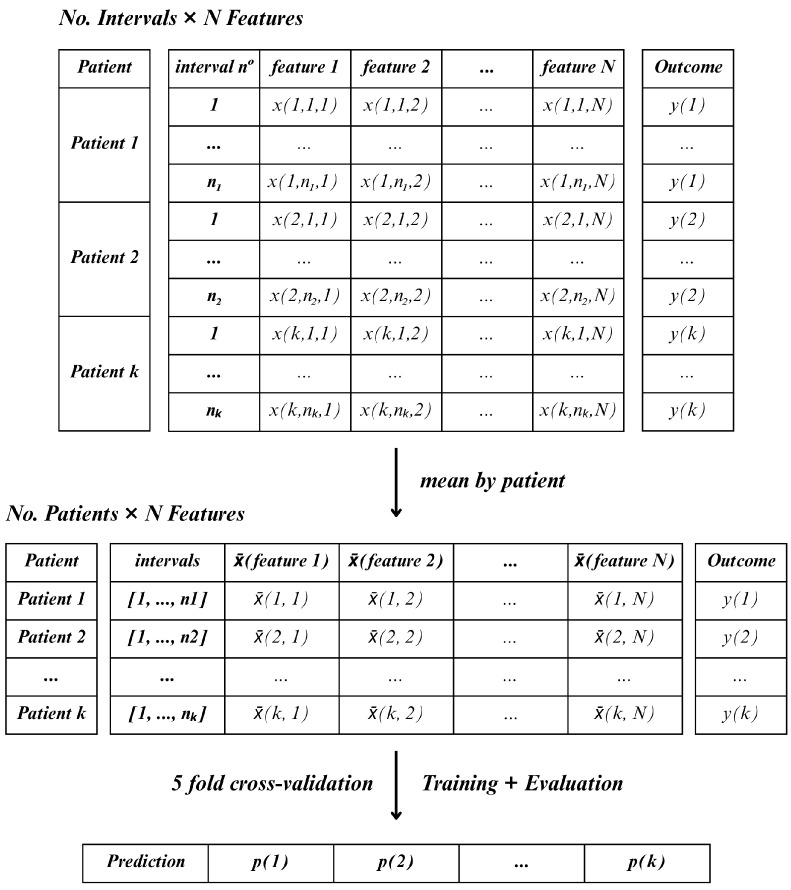
Method D—mean of each feature across all windows.

In contrast, Method D followed a more compressed, patient-level approach. For each lead, the mean value of each feature was calculated across all windows (inter2, inter5, and inter7) and time periods (15, 30, or 60-min). This resulted in one aggregated feature vector per patient, allowing the classifier to operate on a single sample per patient Figure 7. This method reduced the number of samples to match the number of patients.

In comparison with each other, the two methods differ primarily in how they handle temporal variability and feature aggregation. While Method C treats each window as an independent sample, resulting in a higher number of instances per patient and requiring majority voting to determine the final classification, Method D aggregates the features across all windows, producing a single observation per patient. Thus, Method C emphasizes short-term variability within the observation period, whereas Method D captures a more stable, averaged representation of the patient’s signal.

The classification models applied to both Method D and Method C included Linear Discriminant Analysis (LDA), Random Forest (RF), Support Vector Machines (SVM), Gradient Boosting Classifier (GB), and Multilayer Perceptron (MLP) [24]. A 5-fold cross-validation strategy was employed in order to reliably evaluate the performance of these models. To illustrate this, consider a dataset comprising 50 patients, with each split or fold encompassing 10 patients. The model is trained using 80% of the data from each fold and tested on the remaining 20%. Since Method C retains the feature observations across the various windows, the number of rows in each fold may vary. Furthermore, the model will predict life or death for each instance independently, and then the final prediction result is determined by the most frequent prediction among the instances of each patient.

In relation to the metrics employed for the evaluation of the classification outcomes, it is imperative to acknowledge the imbalanced nature of the dataset in this study. The dataset exhibits a preponderance of negative instances, a circumstance that has the potential to engender erroneous metrics, such as accuracy, if the model is predominantly predicting negative outcomes. To this end, the ensuing measures have been utilized for the purpose of evaluating the efficacy of the prediction model:

(1) $$Precision=\frac{TP}{TP+FP}$$

(2) $$Recall=\frac{TP}{TP+FN}$$

(3) $$F_{1}=\frac{2\cdot Precision\cdot Recall}{Precision+Recall}=\frac{2*TP}{2*TP+FP+FN}$$

(4) $$Accuracy=\frac{TP+TN}{TP+TN+FP+FN}$$

(5) AUC

where TP: True positive cases;

TN: True negative cases;
FP: False positive cases;
FN: False negative cases.

The precision or the positive predictive value is the proportion of true positive predictions among all positive predictions (Equation (1)). The recall or the true positive rate is defined as the proportion of positive data points that are correctly identified as positive with respect to all data points that are positive. The calculation of this indicator involves the ratio of correct predictions to the total number of input samples (Equation (2)). The F1-score can be defined as the harmonic mean between recall and precision, which is expressed in the range [0,1]. This metric is typically indicative of the precision (i.e., the accuracy with which instances are classified) and robustness (i.e., the ability to detect significant instances) of the classifier (Equation (3)). The receiver operation curve (ROC), which plots true positive rate versus false positive rate at various thresholds, was used to estimate the area under the ROC—(AUC)—which measures the overall ability of the model to distinguish between the two classes of the outcome.

## 3. Results

The performance of the classification models was evaluated across both Method C and Method D for three different time periods of signal observation 15, 30, and 60 min. The evaluation was based on 5-fold cross-validation, and the main performance metrics considered were F1 and AUC (mean and standard deviation $\bar{F1}\;\pm$ Std and $\bar{AUC}\;\pm$ Std), although recall (mean and standard deviation $\bar{Rec}\;\pm$ Std) and precision (mean and standard deviation $\bar{Prec}\;\pm$ Std) were also computed for deeper analysis. The classification accuracy (mean and standard deviation $\bar{Acc}\;\pm$ Std) was computed in some cases, and the confusion matrix accumulated through the several folds is also shown, with TN, FP, FN, and TP denoting the true negative, false positive, false negative, and true positive values, respectively.

### 3.1. Comparison Between Methods

To understand the effect of feature aggregation strategies, a comparative analysis was performed between Method C (all windows as independent instances with majority voting) and Method D (mean aggregation across windows per patient). As summarized in Table 4, this comparison was performed using a single ECG lead (lead I) across all classification methods, varying observation time periods (T) of 15, 30, and 60 min and window lengths of 2, 5, and 7 min—represented by inter2, inter5, and inter7, respectively.

The results indicate that Method D consistently outperformed Method C, especially in terms of the F1-score, across nearly all configurations. This suggests that the averaging strategy adopted in Method D may provide a more robust and noise-resistant representation of HRV dynamics for patient-level classification, even when using a relatively simple linear classifier such as LDA.

Notably, Method C showed higher variance and, in several cases, reduced performance—likely due to the increased number of instances per patient introducing intra-patient variability and diluting relevant patterns during the voting process. This reinforces the idea that temporal aggregation can enhance model generalization, particularly in clinical settings where interpretability and reliability are critical.

### 3.2. Impact of Time Interval

To evaluate the influence of the observation time period on the classification performance, we show the results for ECG lead I using the full set of HRV features (without feature selection), a constant window size of 5 min, and a single classifier model—LDA. Table 5 presents the results obtained with Method C and Method D across three observation time periods: 15, 30, and 60 min.

The results show that Method D clearly benefited from longer observation periods, with progressive improvements in classification metrics, especially for F1-score and precison. This suggests that averaging the HRV features over a longer monitoring time period enhances signal stability and strengthens the representation of the patient’s physiological state, which improves mortality prediction.

In contrast, Method C did not show the same consistent improvement. The variation in performance across different time periods was less pronounced and, in some cases, unstable, which was likely due to the increased number of samples per patient introducing greater intra-class variability and sensitivity to transient fluctuations in the signal.

These results indicate that when using a per-patient feature aggregation strategy (Method D), extending the initial observation time period from 15 to 60 min can significantly enhance model performance. However, for strategies relying on per-window classification with voting (Method C), the benefit of longer observation time periods is less clear and may depend on additional factors such as feature robustness or window quality.

### 3.3. Model Comparison

To evaluate the impact of the classification algorithm on prediction performance, we conducted a comparative analysis using ECG lead III, the full set of HRV features (without feature reduction), and Method D for feature aggregation. The feature extraction was performed with 5 min windows, and the classification models were tested across three observation time periods—15, 30, and 60 min from the beginning of ICU admission. The models under comparison included LDA, RF, SVM, GB, and MLP.

The results, summarized in Table 6, reveal that GB consistently achieved the highest F1-scores across all observation intervals, which was followed by RF, confirming the effectiveness of ensemble learning approaches in handling complex and redundant feature sets derived from HRV.

Despite not being among the top performers in terms of raw scores, LDA exhibited notable consistency across time intervals, with relatively stable performance. This suggests a degree of robustness, which can be advantageous in clinical contexts that prioritize model interpretability and reliability. In contrast, the SVM model failed to identify any true positives (TP = 0) in this setting, resulting in an F1-score of zero. This outcome highlights a critical limitation: Although SVM may perform well in other contexts, in this scenario, it lacked the sensitivity needed to detect high-risk patients. Such behavior significantly undermines its usefulness in mortality prediction tasks, where minimizing false negatives is crucial. The MLP classifier showed intermediate performance, particularly improving with shorter observation intervals, but it also exhibited greater variability, possibly due to the limited sample size and the model’s sensitivity to parameter tuning.

These findings underscore the superior performance of ensemble classifiers, particularly GB, and suggest caution when applying SVM to unbalanced clinical datasets, especially when the positive class (deceased) is critical to identify.

### 3.4. Feature Reduction Strategies

Given the high dimensionality of the HRV feature set (83 features), we evaluated the impact of feature reduction on model performance. The goal was to assess whether reducing the number of features could improve classification accuracy and generalizability and to identify the most relevant HRV indices for predicting ICU mortality. The evaluated feature reduction techniques included variance thresholding (VT), K-best selection (KB), tree-based selection (SB), and SHAP-based selection (SH).

The results, presented in Table 7, show that for LDA, all feature reduction methods improved performance, consistently increasing the F1-score across all time intervals, suggesting that LDA, being a linear model, benefits significantly from a compact feature set. In contrast, for GB and RF, the effect of feature reduction was more nuanced. While some reduced feature sets led to slight improvements in F1-score, the gains were not consistent across all selection methods, and in some cases, the reduced feature sets even led to slightly lower F1-scores.

This behavior aligns with the nature of tree-based methods, which are inherently robust to irrelevant features due to their internal structure and embedded feature selection. These findings highlight that the impact of feature selection is model-dependent. While linear models such as LDA can greatly benefit from reducing noise and redundancy in the input data, ensemble models like GB are capable of handling high-dimensional feature spaces without requiring aggressive pre-processing, though they may still benefit in some cases from targeted feature selection.

## 4. Discussion

This study focused on exploring the potential of HRV indices as predictors of ICU mortality in patients with COVID-19. HRV indices were extensively extracted from the time domain (19 features), frequency domain (8 features), and nonlinear domain (56 features), resulting in a high-dimensional dataset. To manage this complexity and evaluate predictive performance, two feature selection methods, four feature reduction methodologies, and five classification models were employed. When comparing feature selection strategies, aggregating feature means across patient windows (Method D) demonstrated superior results, especially over longer time periods. In terms of model classifiers, LDA was the algorithm that produced more consistent results, offering stable performance across different windows lengths and improving further with feature reduction. On the other hand, GB and RF also achieved very good F1-scores but were not so consistent across all time intervals.

The effectiveness of LDA concerning models involving HRV features was already demonstrated in previous studies. For instance, in the study by Chen et al. [25], LDA was the best-performing model among the 15 models tested for predicting mortality in 2343 ICU patients with heart failure, achieving the highest accuracy, recall, and F1-score. A web-based calculator was even developed based on this LDA model to make predictions regarding survival, using 44 features (including clinical data and HRV features). GB performed comparably well, while SVM showed the weakest performance, similarly to our study’s findings.

Similarly, in a study by Chiew et al. [26], HRV features were combined with clinical data in order to develop machine learning models to predict 30-day hospital mortality in 214 sepsis patients from the emergency department. Those based on GB outperformed other machine learning approaches as well as conventional scoring systems like the Quick Sequential Organ Failure Assessment, National Early Warning Score, and Modified Early Warning Score.

In our study, analyzing the most frequently selected features in all models and reduction methods, several indices consistently emerged. These include the minimum interval between beats (MinNN), Very High Frequency (VHF), Approximate Entropy (ApEn), Katz Fractal Dimension (KFD), and several complexity and entropy measures such as Multiscale Entropy (MSEn), Composite Multiscale Entropy (CMSEn), Fuzzy Entropy, Shannon Entropy, Lempel–Ziv Complexity (LZC), and Correlation Dimension (CD). The prominence of nonlinear and complexity-based features suggests that the dynamic and fractal properties of HRV provide valuable information about patients’ physiological states according to the study by Sassi et al. [27].

When focusing specifically on the two best-performing combinations (with feature selection) in terms of F1-score—K-best with LDA and SHAP-based feature selection with LDA—additional discriminative features were identified, including the mean and median of NN intervals (MeanNN, MedianNN), percentile-based measures (Prc20NN, Prc80NN) and multifractal indices from detrended fluctuation analysis. Notably, twelve features were shared across both combinations, including MinNN, VHF, ApEn, Shannon Entropy, Fuzzy Entropy, MSEn, CMSEn, CD, KFD, and LZC.

**Figure 8:**
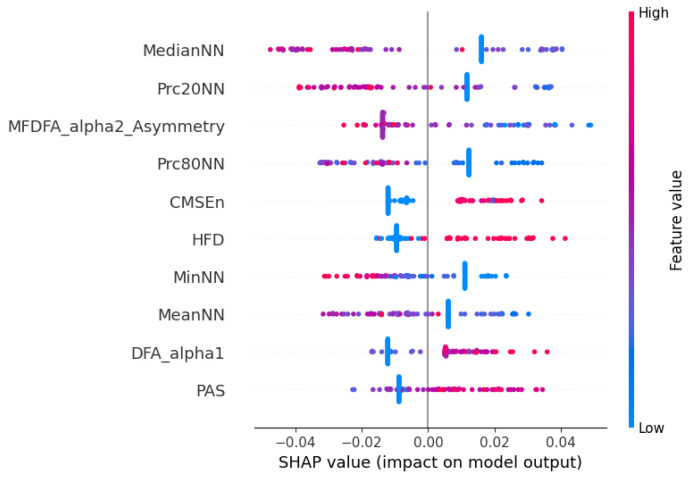
Beeswarm plot illustrating the contribution of each HRV feature to the model’s output based on SHAP values. Each dot represents a data instance, with color indicating the normalized feature value (blue = low, red = high), and the position on the *x* axis representing the SHAP value (impact on model prediction). Positive SHAP values indicate a contribution toward the predicted class, while negative values indicate the opposite.

These findings are further supported by the SHAP beeswarm plot shown in Figure 8, representing the individual contribution of each HRV feature to the model’s prediction. The vertical axis displays the different features, while the horizontal axis shows the SHAP value, indicating how much each feature value contributes to the deviation from the model’s average output. Each dot corresponds to one observation in the dataset, and its color reflects the magnitude of the feature value (blue for low values and red for high values). Consistent with the previous selection analysis, features such as MedianNN, Prc20NN, and MeanNN emerged as highly influential, with higher values positively impacting the model output. Additionally, nonlinear features like DFA-alpha1, HFD, and CMSEn also demonstrated substantial contributions, reinforcing the importance of signal complexity and fractal properties. Interestingly, lower values of DFA-alpha1 and HFD were generally associated with negative SHAP values, potentially indicating that reduced complexity in RR dynamics correlates with a greater likelihood of hospital discharge.

### Limitations

Despite the promising results obtained in this study, several limitations must be acknowledged. First, the dataset used was collected in a single hospital during the COVID-19 pandemic, which may limit the generalizability of the findings to other institutions or to non-COVID-19 ICU populations. Hence, future studies involving larger, multicenter cohorts are necessary to validate the obtained results. Moreover, while the raw data provided a valuable real-world scenario, they presented considerable variability in structure, signal quality, and annotation consistency, requiring extensive pre-processing and data curation. Although this was addressed through a robust pipeline, some signal loss and patient exclusions were inevitable.

Second, the prediction models relied exclusively on ECG-derived HRV features. While HRV captures important aspects of autonomic regulation, incorporating other physiological signals (e.g., respiratory rate, $SpO_{2}$, blood pressure) or clinical variables (e.g., age, comorbidities, laboratory results) might improve predictive performance and allow for a more comprehensive risk assessment, and in the future, we will perform data fusion. Furthermore, due to limited access to complete clinical data for some patients, it was only possible to confirm after the analysis that approximately 5% had a history of ischemic heart disease and around 6% had heart failure. Although no patients with documented severe arrhythmias or malignant rhythm disturbances were included, it is very difficult to be entirely certain given the missing information in some patient records. Therefore, while some influence on HRV from underlying cardiovascular disease cannot be entirely ruled out, its overall impact on our findings is likely limited. Additionally, HRV is sensitive to noise, ectopic beats, and other abnormal cardiac events, which are more frequent in ICU patients. Although R-peak detection and basic artifact removal were applied, residual artifacts may still have affected feature quality.

Third, the analysis of HRV itself presents intrinsic limitations. HRV measures assume stationarity within the analysis windows—an assumption that may not always hold in critically ill patients. Furthermore, although short windows (2 to 7 min) were chosen to match early prediction goals, some nonlinear or frequency-domain features are known to be less reliable in short segments. Several features had to be discarded in some cases due to insufficient data points, which affected the consistency of the features across samples. Additionally, HRV is sensitive to noise and ectopic beats, which are more frequent in ICU patients; although R-peak detection and basic artifact removal were applied, residual artifacts may still have affected feature quality.

Fourth, the sample size—although reasonable—remains limited, especially when broken down by the lead, time interval, and analysis methods. This affects the statistical power of certain comparisons and increases the risk of overfitting, particularly in more complex models. Future validation in larger and multicentric datasets is essential, as well as validation with data not coming from COVID-19.

Finally, although the study focused on early prediction (within 15, 30, and 60 min of ICU admission), the temporal evolution of HRV during the hospitalization was not considered. Analyzing HRV dynamics over time could offer additional insights into patient deterioration or recovery patterns, and sequence analysis with Recurrent Neunral Networks (RNNs), such as Long Short-Term Memory (LSTM), is planned in the future.

## 5. Conclusions

This work was developed based on a real-world database collected during the COVID-19 pandemic, where the raw data posed significant challenges in terms of structure, synchronization, and signal quality. The ability to work with such complex and heterogeneous data is itself a key contribution of this study, highlighting the importance of robust pre-processing pipelines in clinical data analysis in challenging conditions, such as during COVID-19 pandemic.

We explored whether short-term HRV features, extracted from ECG signals recorded during the very first period of ICU admission, could be used to predict patient mortality. This question is of high clinical relevance, since early identification of high-risk patients enables clinicians to adapt therapeutic strategies, potentially improving outcomes in intensive care settings. In pandemic contexts such as COVID-19, where ICU resources are limited and patient deterioration can be rapid, such predictive tools cloud have a significant impact on clinical decision making.

Our results show that meaningful mortality prediction can be achieved using only the first 15 to 60 min of ECG data. Method D, which aggregates HRV features across short windows, demonstrated superior performance compared to treating each time window individually. Among the classification models tested, GB and RF achieved the highest F1-scores. However, LDA stood out for its remarkable stability and consistent performance across different leads, time intervals, and feature selection strategies—often ranking among the top-performing models. This reinforces its potential value as a clinically interpretable and computationally efficient approach.

Feature reduction techniques also played an important role, particularly when combined with LDA, where they contributed to enhanced performance. In contrast, more complex models like GB benefited less consistently from dimensionality reduction.

Overall, this study reinforces the potential of HRV analysis as a noninvasive, low-cost, and readily available tool for early mortality risk stratification in the ICU. Future work will aim to expand this approach to larger datasets, include additional physiological signals, and move toward the development of real-time, clinically integrated decision support systems.

## Figures and Tables

**Table 1:** Time-domain, frequency-domain, and nonlinear HRV features.

Time Domain	Frequency Domain	Nonlinear	
MeanNN	LF	SD1	DFA alpha1 Width
SDNN	HF	SD2	DFA alpha1 Peak
RMSSD	VHF	SD1/SD2	DFA alpha1 Mean
SDSD	TP	S	DFA alpha1 Max
CVNN	LFHF	CSI	DFA alpha1 Delta
CVSD	LFn	CVI	DFA alpha1 Asymmetry
MedianNN	HFn	CSI_Modified	DFA alpha1 Fluctuation
MadNN	LnHF	GI	DFA alpha1 Increment
MCVNN		SI	DFA alpha2
IQRNN		AI	DFA alpha2 Width
SDRMSSD		PI	DFA alpha2 Peak
Prc20NN		SD1d	DFA alpha2 Mean
Prc80NN		SD1a	DFA alpha2 Max
pNN50		C1d	DFA alpha2 Delta
pNN20		C1a	DFA alpha2 Asymmetry
MinNN		SD2d	DFA alpha2 Fluctuation
MaxNN		SD2a	DFA alpha2 Increment
HTI		C2d	ApEn
TINN		C2a	SampEn
		SDNNd	ShanEn
		SDNNa	FuzzyEn
		Cd	MSEn
		Ca	CMSEn
		PIP	RCMSEn
		IALS	CD
		PSS	HFD
		PAS	KFD
		DFA alpha1	LZC

**Table 2:** Demographic and clinical baseline characteristics of patients selected for HRV analysis. Continuous variables are presented as median (25th percentile–75th percentile) and categorical variables as number (n) and percentage (%). For variables with missing data, the number of observations used (n) is indicated. Abbreviations: ECMO—extracorporeal membrane oxygenation.

	Overall (N = 82, 100%)
**Age, years**	57.00 (44.00–71.00)
**Age category**	
<60 years	44 (53.7)
≥60 years	38 (46.3)
**Gender**	
Female	28 (34.1)
Male	54 (65.9)
**BMI, Kg/m^2^**	27.68 (25.09–29.40), n = 77
**Geographic origin**	
Portugal	64 (78.0)
Other European countries	5 (6.1)
Africa	6 (7.3)
America	3 (3.7)
Asia	4 (4.9)
**COVID-19 wave**	
First	3 (3.7)
Second	15 (18.3)
Third	24 (29.3)
Fourth	27 (32.9)
Fifth	13 (15.9)
**Number of comorbidities**	
0	28 (34.1)
1	13 (15.9)
2	12 (14.6)
3	15 (18.3)
4	8 (9.8)
5	3 (3.7)
6	3 (3.7)
**Kinds of comorbidities**	
Arterial hypertension	34 (41.5)
Diabetes	16 (19.5)
Dyslipidemia	20 (24.4)
Ischemic heart disease	4 (4.9)
Heart failure	5 (6.1)
Stroke	3 (3.7)
Chronic respiratory disease	6 (7.3)
Chronic renal disease	9 (11.0)
Chronic liver disease	3 (3.7)
Solid cancer	5 (6.1)
Hematologic cancer	2 (2.4)
Autoimmune disease	3 (3.7)
Hypothyroidism	8 (9.8)
Obesity	26 (31.7)
**Respiratory support**	
Invasive mechanical ventilation	52 (63.4)
ECMO	20 (24.4)
**Admission motive**	
Respiratory failure / insufficiency	39 (47.6)
Sepsis	12 (14.6)
Shock	5 (6.1)
Neurological impairment	4 (4.9)
Renal dysfunction	1 (1.2
Cardiac arrest	3 (3.7)
Polytrauma	2 (2.4)
Other reasons	16 (19.5)
**ICU length of stay, days**	6.00 (3.00–11.50)
**ICU outcome**	
Discharged	66 (80.5)
Deceased	16 (19.5)

**Table 3:** Distribution of patients per ECG lead.

Lead	Total	Discharges	Deaths
**ECG I**	64	51	13
**ECG II**	77	61	16
**ECG III**	60	47	13
**ECG AVF**	68	53	15

**Table 4:** Results for ECG lead I, comparing performance of Method C and Method D, all models, across varying observation time periods (15, 30, and 60 min), and window lengths (2, 5, and 7 min). Results are sorted by F1-score. Abbreviations: Red—feature reduction method; inter—interval; T—time period; M—method; $\bar{Pre}\;\pm$ Std—precision mean and standard deviation; $\bar{Rec}\;\pm$ Std—recall mean and standard deviation; $\bar{F1}\;\pm$ Std—F1-score mean and standard deviation; $\bar{AUC}\;\pm$ Std—AUC mean and standard deviation; TN—true negative; FP—false positive; FN—false negative; TP—true positive.

Red	Inter	T	M	Model	$\bar{\mathrm{Prec}}\;\pm$ Std	$\bar{\mathrm{Rec}}\;\pm$ Std	$\bar{F1}\;\pm$ F1 Std	$\bar{\mathrm{AUC}}\;\pm$ Std	TN	FP	FN	TP
All	inter5	60	D	LDA	0.52 ± 0.11	0.80 ± 0.25	0.61 ± 0.14	0.82 ± 0.13	44	8	3	9
VT	inter7	60	D	LDA	0.43 ± 0.13	0.68 ± 0.29	0.51 ± 0.17	0.74 ± 0.17	42	10	5	7
VT	inter2	30	D	LDA	0.38 ± 0.12	0.70 ± 0.25	0.47 ± 0.13	0.71 ± 0.16	38	14	4	8
VT	inter7	30	D	LDA	0.35 ± 0.20	0.70 ± 0.40	0.45 ± 0.25	0.74 ± 0.19	40	12	4	8
VT	inter2	60	D	GB	0.53 ± 0.40	0.52 ± 0.41	0.45 ± 0.32	0.72 ± 0.20	48	4	7	5
SB	inter7	30	D	LDA	0.37 ± 0.25	0.55 ± 0.40	0.44 ± 0.30	0.70 ± 0.22	44	8	6	6
SB	inter5	60	D	GB	0.37 ± 0.31	0.53 ± 0.45	0.43 ± 0.36	0.72 ± 0.22	47	5	6	6
SH	inter7	60	D	LDA	0.45 ± 0.28	0.52 ± 0.26	0.42 ± 0.14	0.67 ± 0.15	43	9	7	5
VT	inter5	60	D	MLP	0.50 ± 0.45	0.37 ± 0.37	0.41 ± 0.39	0.66 ± 0.21	49	3	7	5
All	inter5	60	C	MLP	0.80 ± 0.40	0.28 ± 0.16	0.41 ± 0.22	0.63 ± 0.10	50	1	9	4
All	inter2	60	D	LDA	0.40 ± 0.23	0.43 ± 0.23	0.41 ± 0.21	0.63 ± 0.13	43	9	6	6
SH	inter5	60	D	GB	0.33 ± 0.28	0.53 ± 0.45	0.41 ± 0.34	0.71 ± 0.21	46	6	6	6
SB	inter2	15	D	LDA	0.43 ± 0.33	0.43 ± 0.23	0.40 ± 0.22	0.63 ± 0.09	43	9	6	6
All	inter5	60	D	MLP	0.47 ± 0.45	0.37 ± 0.37	0.40 ± 0.39	0.65 ± 0.21	48	4	7	5
VT	inter5	60	D	LDA	0.36 ± 0.34	0.47 ± 0.32	0.40 ± 0.33	0.60 ± 0.22	38	14	6	6
KB	inter2	60	D	LDA	0.48 ± 0.32	0.42 ± 0.33	0.39 ± 0.23	0.64 ± 0.15	45	7	7	5
SH	inter5	60	C	MLP	0.60 ± 0.49	0.30 ± 0.27	0.39 ± 0.34	0.64 ± 0.14	50	1	9	4
KB	inter5	60	D	GB	0.42 ± 0.38	0.40 ± 0.37	0.38 ± 0.32	0.66 ± 0.17	48	4	7	5

**Table 5:** Results for ECG lead I, comparing the performance of time period, with no feature reduction, and LDA model, evaluated with Method C and Method D, with a fixed window length of 5 min. Abbreviations: T—analyzed time period; M—method; $\bar{Pre}\;\pm$ Std—precision mean and standard deviation; $\bar{Rec}\;\pm$ Std—recall mean and standard deviation; $\bar{F1}\;\pm$ Std—F1-score mean and standard deviation; $\bar{AUC}\;\pm$ Std—AUC mean and standard deviation; TN—true negative; FP—false positive; FN—false negative; TP—true positive.

T	M	$\bar{\mathrm{Prec}}\;\pm$ Std	$\bar{\mathrm{Rec}}\;\pm$ Std	$\bar{F1}\;\pm$ Std	$\bar{\mathrm{AUC}}\;\pm$ Std	TN	FP	FN	TP
60	D	0.52 ± 0.11	0.80 ± 0.25	0.61 ± 0.14	0.82 ± 0.13	44	8	3	9
30	D	0.28 ± 0.27	0.40 ± 0.37	0.29 ± 0.25	0.60 ± 0.21	41	11	8	4
15	D	0.16 ± 0.14	0.35 ± 0.37	0.19 ± 0.16	0.50 ± 0.22	34	18	9	3
60	C	0.30 ± 0.40	0.20 ± 0.27	0.24 ± 0.32	0.58 ± 0.14	49	2	10	3
30	C	0.37 ± 0.37	0.18 ± 0.15	0.23 ± 0.19	0.55 ± 0.06	47	4	10	3
15	C	0.12 ± 0.15	0.12 ± 0.15	0.11 ± 0.14	0.46 ± 0.05	41	10	11	2

**Table 6:** Results for ECG lead III, comparing performance of classification models, without feature reduction, using a fixed window length of 5 min, with Method D for feature aggregation, across three observation time periods (15, 30, and 60 min). For each model, the results are sorted by time period. Abbreviations: W—time period; M—method; $\bar{Acc}\;\pm$ Std—accuracy mean and standard deviation; $\bar{Pre}\;\pm$ Std—precision mean and standard deviation; $\bar{Rec}\;\pm$ Std—recall mean and standard deviation; $\bar{F1}\;\pm$ Std—F1-score mean and standard deviation; TN—true negative; FP—false positive; FN—false negative; TP—true positive.

Model	W	M	$\bar{\mathrm{Acc}}\;\pm$ Std	$\bar{\mathrm{Pre}}\;\pm$ Std	$\bar{\mathrm{Rec}}\;\pm$ Std	$\bar{F1}\;\pm$ Std	TN	FP	FN	TP
GB	15	D	0.85 ± 0.10	0.63 ± 0.37	0.57 ± 0.39	0.53 ± 0.28	46	2	7	5
GB	30	D	0.77 ± 0.10	0.18 ± 0.22	0.25 ± 0.39	0.18 ± 0.23	43	5	9	3
GB	60	D	0.73 ± 0.03	0.30 ± 0.37	0.35 ± 0.37	0.23 ± 0.19	41	7	9	3
LDA	15	D	0.70 ± 0.07	0.25 ± 0.13	0.42 ± 0.33	0.28 ± 0.15	38	10	8	4
LDA	30	D	0.78 ± 0.09	0.33 ± 0.28	0.32 ± 0.37	0.31 ± 0.30	43	5	8	4
LDA	60	D	0.70 ± 0.07	0.18 ± 0.15	0.35 ± 0.37	0.22 ± 0.18	39	9	9	3
MLP	15	D	0.77 ± 0.06	0.28 ± 0.39	0.27 ± 0.39	0.21 ± 0.26	43	5	9	3
MLP	30	D	0.80 ± 0.09	0.33 ± 0.42	0.27 ± 0.39	0.26 ± 0.33	45	3	9	3
MLP	60	D	0.75 ± 0.09	0.00 ± 0.00	0.00 ± 0.00	0.00 ± 0.00	45	3	12	0
RF	15	D	0.82 ± 0.11	0.47 ± 0.45	0.37 ± 0.37	0.38 ± 0.37	46	2	9	3
RF	30	D	0.77 ± 0.06	0.20 ± 0.40	0.05 ± 0.10	0.08 ± 0.16	45	3	11	1
RF	60	D	0.80 ± 0.07	0.20 ± 0.40	0.05 ± 0.10	0.08 ± 0.16	47	1	11	1
SVC	15	D	0.80 ± 0.09	0.00 ± 0.00	0.00 ± 0.00	0.00 ± 0.00	48	0	12	0
SVC	30	D	0.80 ± 0.09	0.00 ± 0.00	0.00 ± 0.00	0.00 ± 0.00	48	0	12	0
SVC	60	D	0.80 ± 0.09	0.00 ± 0.00	0.00 ± 0.00	0.00 ± 0.00	48	0	12	0

**Table 7:** Comparative performance across feature reduction strategies—using ECG lead III and a 30 min observation window, with a fixed interval of 2 min; Method D for feature aggregation; and classification models LDA, RF, and GB. Abbreviations: Red—feature reduction method; $\bar{Pre}\;\pm$ Std—precision mean and standard deviation; $\bar{Rec}\;\pm$ Std—recall mean and standard deviation; $\bar{F1}\;\pm$ Std—F1-score mean and standard deviation; $\bar{AUC}\;\pm$ Std—AUC mean and standard deviation; TN—true negative; FP—false positive; FN—false negative; TP—true positive.

Red	Model	$\bar{\mathrm{Prec}}\;\pm$ Std	$\bar{\mathrm{Rec}}\;\pm$ Std	$\bar{F1}\;\pm$ Std	$\bar{\mathrm{AUC}}\;\pm$ Std	TN	FP	FN	TP
All	LDA	0.13 ± 0.19	0.30 ± 0.40	0.16 ± 0.20	0.52 ± 0.19	35	13	10	2
All	RF	0.33 ± 0.42	0.25 ± 0.39	0.24 ± 0.32	0.61 ± 0.18	46	2	9	3
All	GB	0.23 ± 0.29	0.25 ± 0.39	0.23 ± 0.31	0.58 ± 0.19	44	4	9	3
KB	LDA	0.63 ± 0.31	0.52 ± 0.26	0.50 ± 0.15	0.70 ± 0.15	43	5	7	5
KB	RF	0.83 ± 0.21	0.62 ± 0.32	0.66 ± 0.23	0.79 ± 0.17	46	2	6	6
KB	GB	0.43 ± 0.39	0.45 ± 0.46	0.43 ± 0.41	0.68 ± 0.25	44	4	8	4
SB	LDA	0.63 ± 0.31	0.52 ± 0.26	0.48 ± 0.11	0.71 ± 0.12	43	5	7	5
SB	RF	0.53 ± 0.32	0.55 ± 0.40	0.49 ± 0.29	0.74 ± 0.19	45	3	7	5
SB	GB	0.33 ± 0.42	0.25 ± 0.39	0.24 ± 0.32	0.60 ± 0.19	45	3	9	3
SH	LDA	0.52 ± 0.41	0.47 ± 0.32	0.39 ± 0.22	0.66 ± 0.16	41	7	8	4
SH	RF	0.40 ± 0.37	0.35 ± 0.37	0.33 ± 0.30	0.65 ± 0.18	46	2	9	3
SH	GB	0.40 ± 0.39	0.30 ± 0.25	0.33 ± 0.28	0.61 ± 0.11	44	4	8	4
VT	LDA	0.15 ± 0.18	0.30 ± 0.40	0.19 ± 0.23	0.51 ± 0.17	34	14	8	4
VT	RF	0.00 ± 0.00	0.00 ± 0.00	0.00 ± 0.00	0.49 ± 0.02	47	1	12	0
VT	GB	0.30 ± 0.40	0.15 ± 0.20	0.20 ± 0.27	0.53 ± 0.12	44	4	10	2

## Data Availability

The datasets used for this study are available from the corresponding author upon reasonable request.

## Supplementary Materials

The following supporting information can be downloaded at: https://www.mdpi.com/article/10.3390/jcm14155312/s1, Table S1: Description of the time-domain indices of HRV; Table S2: Description of the frequency domain indices of HRV; Table S3: Description of the basic indices derived from the Poincaré plot analysis; Table S4: Description for the Indices of Heart Rate Asymmetry; Table S5: Description of the Indices of Heart Rate Fragmentation; Table S6: Description of the Indices of Complexity and Fractal Physiology; Table S7: Description of the Indices of Complexity and Fractal Physiology.

## References

[1] Agabiti-Rosei E., Huhtaniemi I., Martini L. (2019). Antiadrenergic Agents. Encyclopedia of Endocrine Diseases.

[2] Vaseghi M., Shivkumar K. (2008). The role of the autonomic nervous system in sudden cardiac death. Prog. Cardiovasc. Dis..

[3] Shaffer F., Ginsberg J.P. (2017). An Overview of Heart Rate Variability Metrics and Norms. Front. Public Health.

[4] Jarczok M.N., Weimer K., Braun C., Williams D.P., Thayer J.F., Gündel H.O., Balint E.M. (2022). Heart rate variability in the prediction of mortality: A systematic review and meta-analysis of healthy and patient populations. Neurosci. Biobehav. Rev..

[5] Tiwari R., Kumar R., Malik S., Raj T., Kumar P. (2021). Analysis of Heart Rate Variability and Implication of Different Factors on Heart Rate Variability. Curr. Cardiol. Rev..

[6] Hejjel L., Gál I. (2001). Heart rate variability analysis. Acta Physiol. Hung..

[7] Costa M.D., Davis R.B., Goldberger A.L. (2017). Heart Rate Fragmentation: A New Approach to the Analysis of Cardiac Interbeat Interval Dynamics. Front. Physiol..

[8] Piskorski J., Guzik P. (2011). Asymmetric properties of long-term and total heart rate variability. Med. Biol. Eng. Comput..

[9] Makowski D., Pham T., Lau Z.J., Brammer J.C., Lespinasse F., Pham H., Schölzel C., Chen S.H.A. (2021). NeuroKit2: A Python toolbox for neurophysiological signal processing. Behav. Res. Methods.

[10] Bishop D.G., Wise R.D., Lee C., von Rahden R.P., Rodseth R.N. (2016). Heart rate variability predicts 30-day all-cause mortality in intensive care units. S. Afr. J. Anaesth. Analg..

[11] Moridani M.K., Setarehdan S.K., Nasrabadi A.M., Hajinasrollah E. (2015). Analysis of heart rate variability as a predictor of mortality in cardiovascular patients of intensive care unit. Biocybern. Biomed. Eng..

[12] Liu N., Chee M.L., Foo M.Z.Q., Pong J.Z., Guo D., Koh Z.X., Ho A.F.W., Niu C., Chong S.L., Ong M.E.H. (2021). Heart rate n-variability (HRnV) measures for prediction of mortality in sepsis patients presenting at the emergency department. PLoS ONE.

[13] Komaenthammasophon C., Pachinburavan M., Chokesuwattanaskul R. (2024). Heart rate variability and mortality in critically ill COVID-19 pneumonia patients. Heliyon.

[14] Mol M.B.A., Strous M.T.A., van Osch F.H.M., Vogelaar F.J., Barten D.G., Farchi M., Foudraine N.A., Gidron Y. (2021). Heart-rate-variability (HRV), predicts outcomes in COVID-19. PLoS ONE.

[15] Dumargne H., Patural H., Charbonnieras F., Charier D., Biscarrat C., Chivot M., Argaud L., Cour M., Dargent A. (2024). Exploration of COVID-19 associated bradycardia using heart rate variability analysis in a case-control study of ARDS patients. Heart Lung J. Cardiopulm. Acute Care.

[16] Bento L., Fonseca-Pinto R., Póvoa P. (2017). Autonomic nervous system monitoring in intensive care as a prognostic tool. Systematic review. Rev. Bras. Ter. Intensiv..

[17] Von Rekowski C.P., Pinto I., Fonseca T.A.H., Araújo R., Calado C.R.C., Bento L. (2025). Analysis of six consecutive waves of ICU-admitted COVID-19 patients: Key findings and insights from a Portuguese population. GeroScience.

[18] Pedregosa F., Varoquaux G., Gramfort A., Michel V., Thirion B., Grisel O., Blondel M., Prettenhofer P., Weiss R., Dubourg V. (2011). Scikit-learn: Machine Learning in Python. J. Mach. Learn. Res..

[19] Fortner B. (1998). HDF: The hierarchical data format. Dr. Dobb’s J. Softw. Tools Prof. Program..

[20] Lee H., Yang H.L., Ryu H.G., Jung C.W., Cho Y.J., Yoon S.B., Yoon H.K., Lee H.C. (2023). Real-time machine learning model to predict in-hospital cardiac arrest using heart rate variability in ICU. NPJ Digit. Med..

[21] Hamilton P.S., Tompkins W.J. (1986). Quantitative investigation of QRS detection rules using the MIT/BIH arrhythmia database. IEEE Trans. Biomed. Eng..

[22] Louppe G. (2015). Understanding Random Forests: From Theory to Practice. arXiv.

[23] Lundberg S.M., Erion G., Chen H., DeGrave A., Prutkin J.M., Nair B., Katz R., Himmelfarb J., Bansal N., Lee S.I. (2020). From Local Explanations to Global Understanding with Explainable AI for Trees. Nat. Mach. Intell..

[24] Hastie T., Tibshirani R., Friedman J. (2009). The Elements of Statistical Learning: Data Mining, Inference, and Prediction.

[25] Chen A.T., Zhang Y., Zhang J. (2025). Explainable machine learning and online calculators to predict heart failure mortality in intensive care units. ESC Heart Fail..

[26] Chiew C.J., Liu N., Tagami T., Wong T.H., Koh Z.X., Ong M.E.H. (2019). Heart rate variability based machine learning models for risk prediction of suspected sepsis patients in the emergency department. Medicine.

[27] Sassi R., Cerutti S., Lombardi F., Malik M., Huikuri H.V., Peng C., Schmidt G., Yamamoto Y. (2015). Advances in heart rate variability signal analysis: Joint position statement by the e-Cardiology ESC Working Group and the European Heart Rhythm Association co-endorsed by the Asia Pacific Heart Rhythm Society. EP Eur..

