# Supplementary material for: Analyzing Heart Rate Variability for COVID-19 ICU Mortality Prediction Using Continuous Signal Processing Techniques
Source: J Clin Med. 2025 Jul 28;14(15):5312. doi: 10.3390/jcm14155312 (PMC12347825; doi:10.3390/jcm14155312)
Supplement: Supplementary file 1 [file jcm-14-05312-s001.zip › jcm-3716345-supplementary.pdf]

1 Suplemental Material

In the suplemental material we present the tables (S1-S7) defining the used signal features described in Table S1, according Makowski et al. (2021).

Feature	Description
MeanNN	The mean of the RR intervals.
SDNN	The standard deviation of the RR intervals.
RMSSD	The square root of the mean of the squared successive differences between adjacent RR intervals.
SDSD	The standard deviation of the successive differences between RR intervals.
CVNN	The standard deviation of the RR intervals (SDNN) divided by the mean of the RR intervals (MeanNN).
CVSD	The root mean square of successive differences (RMSSD) divided by the mean of the RR intervals (MeanNN).
MedianNN	The median of the RR intervals.
MadNN	The median absolute deviation of the RR intervals.
MCVNN	The median absolute deviation of the RR intervals (MadNN) divided by the median of the RR intervals (MedianNN).
IQRNN	The interquartile range (IQR) of the RR intervals.
SDRMSSD	$SDNN/RMSSD$, a time-domain equivalent for the low Frequency-to-High Frequency (LF/HF) Ratio.
Prc20NN	The 20th percentile of the RR intervals.

Table S1: Description of the time-domain indices of HRV

Feature	Description
LF	The spectral power of low frequencies (by default, .04 to .15 Hz).
HF	The spectral power of high frequencies (by default, .15 to .4 Hz).
VHF	The spectral power of very high frequencies (by default, .4 to .5 Hz).
TP	The total spectral power.
LFHF	The ratio obtained by dividing the low frequency power by the high frequency power.
LFn	The normalized low frequency, obtained by dividing the low frequency power by the total power.
HFn	The normalized high frequency, obtained by dividing the low frequency power by the total power.
LnHF	The log transformed HF.

Table S2: Description of the frequency domain indices of HRV

Feature	Description
SD1	Standard deviation perpendicular to the line of identity.
SD2	Standard deviation along the identity line. Index of long-term HRV changes.
SD1/SD2	Ratio of SD1 to SD2. Describes the ratio of short term to long term variations in HRV.
S	Area of ellipse described by SD1 and SD2 ($pi * SD1 * SD2$). Proportional to SD1SD2.
CSI	The Cardiac Sympathetic Index, a measure of cardiac sympathetic function independent of vagal activity, calculated by dividing he longitudinal variability of the Poincaré plot (4*SD2) by its transverse variability (4*SD1).
CVI	The Cardiac Vagal Index, an index of cardiac parasympathetic function (vagal activity unaffected by sympathetic activity), and is equal equal to the logarithm of the product of longitudinal (4*SD2) and transverse variability (4*SD1).
CSI_Modified	The modified CSI obtained by dividing the square of the longitudinal variability by its transverse variability.

Table S3: Description of the basic indices derived from the Poincaré plot analysis

Feature	Description
GI	Guzik's Index, defined as the distance of points above line of identity (LI) to LI divided by the distance of all points in Poincaré plot to LI except those that are located on LI.
SI	Slope Index, defined as the phase angle of points above LI divided by the phase angle of all points in Poincaré plot except those that are located on LI.
AI	Area Index, defined as the cumulative area of the sectors corresponding to the points that are located above LI divided by the cumulative area of sectors corresponding to all points in the Poincaré plot except those that are located on LI.
PI	Porta's Index, defined as the number of points below LI divided by the total number of points in Poincaré plot except those that are located on LI.
SD1d, SD1a	Short-term variance of contributions of decelerations (prolongations of RR intervals) and accelerations (shortenings of RR intervals), respectively.
C1d, C1a	The contributions of heart rate decelerations and accelerations to short-term HRV, respectively.
SD2d, SD2a	Long-term variance of contributions of decelerations (prolongations of RR intervals) and accelerations (shortenings of RR intervals), respectively.
C2d, C2a	The contributions of heart rate decelerations and accelerations to long-term HRV, respectively.
SDNNd, SDNNa	Total variance of contributions of decelerations (prolongations of RR intervals) and accelerations (shortenings of RR intervals), respectively.
Cd, Ca	The total contributions of heart rate decelerations and accelerations to HRV.

Table S4: Description for the Indices of Heart Rate Asymmetry

Feature	Description
PIP	Percentage of inflection points of the RR intervals series.
IALS	Inverse of the average length of the acceleration/deceleration segments.
PSS	Percentage of short segments.
PAS	Percentage of NN intervals in alternation segments.

Table S5: Description of the Indices of Heart Rate Fragmentation

Feature	Description
DFA alpha1	The monofractal detrended fluctuation analysis (DFA) of the HR signal, corresponding to short-term correlations.
DFA alpha1 Width	The width of the singularity spectrum, which quantifies the degree of multifractality.
DFA alpha1 Peak	The value of the singularity exponent H corresponding to the peak of the singularity dimension D.
DFA alpha1 Mean	The average of the maximum and minimum values of the singularity exponent H, which quantifies the mean fluctuations of the signal.
DFA alpha1 Max	The value of the singularity spectrum D corresponding to the maximum value of the singularity exponent H, indicative of the signal's maximum fluctuation.
DFA alpha1 Delta	The vertical distance in the singularity spectrum D between the minimum and maximum singularity exponents. It corresponds to the range of signal fluctuations.
DFA alpha1 Asymmetry	The Asymmetry Index corresponds to the centrality of the peak of the spectrum.
DFA alpha1 Fluctuation	The fluctuation index h
DFA alpha1 Increment	The cumulative function of the squared increments of generalized Hurst exponents between consecutive moment orders
DFA alpha2	The monofractal detrended fluctuation analysis (DFA) of the HR signal, corresponding to long-term correlations
DFA alpha2 Width	The width of the singularity spectrum, which quantifies the degree of multifractality.
DFA alpha2 Peak	The value of the singularity exponent H corresponding to the peak of the singularity dimension D.
DFA alpha2 Mean	The average of the maximum and minimum values of the singularity exponent H, which quantifies the mean fluctuations of the signal.
DFA alpha2 Max	The value of the singularity spectrum D corresponding to the maximum value of the singularity exponent H, indicative of the signal's maximum fluctuation.
DFA alpha2 Delta	The vertical distance in the singularity spectrum D between the minimum and maximum singularity exponents. It corresponds to the range of signal fluctuations.
DFA alpha2 Asymmetry	The Asymmetry Index corresponds to the centrality of the peak of the spectrum.
DFA alpha2 Fluctuation	The fluctuation index h.
DFA alpha2 Increment	The cumulative function of the squared increments of generalized Hurst exponents between consecutive
ApEn	Approximate Entropy (ApEn), used to quantify the unpredictability of fluctuations in time series data.
SampEn	Sample Entropy (SampEn) of a signal corresponds to the conditional probability that two vectors close to each other in m dimensions will remain close in the next component m+1.
ShanEn	Shannon Entropy of the signal
FuzzyEn	Fuzzy Entropy of the signal

Table S6: Description of the Indices of Complexity and Fractal Physiology

Feature	Description
MSEn	Multiscale Entropy (MSEn), which calculates sample entropies over multiple scales.
CMSEn	Composite Multiscale Entropy, which computes several coarsely refined series for each scale factor.
RCMSEn	Refined Multiscale Entropy, which calculates the average of components at a lower level of entropy values of each sub-grouped vector.
CD	Correlation Dimension is an estimate of the fractal dimension of a signal.
HFD	Higuchi Fractal Dimension is an approximate value of the box-counting dimension for time series.
KFD	Katz Fractal Dimension, corresponding to the average of the sum of Euclidean distances between successive points in the signal and the maximum distance between the starting point and any other point in the sample.
LZC	Lempel-Ziv Complexity, which quantifies complexity by examining symbolic sequences for new patterns, increasing the complexity count whenever a new sequence is detected.

Table S7: Description of the Indices of Complexity and Fractal Physiology
